# Independent of Their Localization in Protein the Hydrophobic Amino Acid Residues Have No Effect on the Molten Globule State of Apomyoglobin and the Disulfide Bond on the Surface of Apomyoglobin Stabilizes This Intermediate State

**DOI:** 10.1371/journal.pone.0098645

**Published:** 2014-06-03

**Authors:** Tatiana N. Melnik, Maria A. Majorina, Daria S. Larina, Ivan A. Kashparov, Ekaterina N. Samatova, Anatoly S. Glukhov, Bogdan S. Melnik

**Affiliations:** 1 Institute of Protein Research, RAS, Pushchino, Moscow Region, Russia; 2 Department of Physical Biochemistry, Max Planck Institute for Biophysical Chemistry, Göttingen, Germany; Aligarh Muslim University, India

## Abstract

At present it is unclear which interactions in proteins reveal the presence of intermediate states, their stability and formation rate. In this study, we have investigated the effect of substitutions of hydrophobic amino acid residues in the hydrophobic core of protein and on its surface on a molten globule type intermediate state of apomyoglobin. It has been found that independent of their localization in protein, substitutions of hydrophobic amino acid residues do not affect the stability of the molten globule state of apomyoglobin. It has been shown also that introduction of a disulfide bond on the protein surface can stabilize the molten globule state. However in the case of apomyoglobin, stabilization of the intermediate state leads to relative destabilization of the native state of apomyoglobin. The result obtained allows us not only to conclude which mutations can have an effect on the intermediate state of the molten globule type, but also explains why the introduction of a disulfide bond (which seems to “strengthen” the protein) can result in destabilization of the protein native state of apomyoglobin.

## Introduction

Myoglobin is a small alpha-helical protein (153 a.a.) chosen by many researchers as a model protein for studying basic principles of self-organization of globular proteins [1]–[4]. At the moment the most studied are the folding and unfolding processes of the apo-form of this protein at different denaturant concentrations, various pH values and temperature [1], [3], [5]–[11]. It was shown that the folding of this protein occurs via the formation of an intermediate state of the molten globule type and can be described using a two-stage sequential scheme of reaction [1], [9], [11]. It was demonstrated using the stopped-flow and quench-flow techniques that urea-induced apomyoglobin refolding goes via a kinetic intermediate that forms within 6 msec and is structurally similar to the equilibrium molten globule intermediate observed at pH 4.2 [9]. Subsequent kinetic studies suggested that this intermediate is on-pathway [12]. Quench-flow amide proton exchange combined with mass-spectrometry confirmed that apomyoglobin folds by a single pathway and that the intermediate is obligatory [13]. In general, the formation of the molten globule state of apomyoglobin proceeds very rapidly during the burst-phase within several msec, which is in the range of the dead time of the instruments use [1], [11]. Therefore, in kinetic experiments it is almost impossible to detect the molten globule state of apomyoglobin. However, the rapidly formed molten globule state affects the parameters characteristic of the following stages of protein folding. The approach that allows taking into account the effect of the fast folding stage of protein folding (occurring during the dead time of the instruments) on the rate of the slow folding stage of this protein is theoretically grounded [1], [14]. This approach permits one to obtain population values of the protein molten globule state versus the denaturant concentration, i.e. to study the stability of the molten globule state, and using the kinetic data of chevron plots it is possible to design the energy landscape of apomyoglobin and explain the peculiarities of the effect of mutations on this protein [1], [11].

The effect of substitutions of different amino acid residues on the native state of apomyoglobin is studied quite well [4], [11], [15]–[16]. Analogous experiments were performed with other proteins as well. The effects of substitutions of different amino acid residues on the stability and the rate of native state formation in various proteins have been studied systematically [17]–[22]. Thus, for some proteins the so-called folding nucleus has been determined with the φ-analysis [22]–[25]. The folding nucleus includes amino acid residues and protein parts the substitutions of which affect the folding/unfolding rate of the protein native state [26]–[32]. In some cases, such studies involve several dozens of mutant proteins with single substitutions of amino acid residues. This permits revealing the localization of residues that determine the rate of formation and the stability of the protein native state. Nevertheless, there are virtually no investigations employing multiple substitutions of amino acid residues for analyzing intermediate states of proteins, although intermediate states of some proteins are functionally important and in many respects determine the properties of proteins. For example, it has been shown that in the molten globule state, proteins are transported via the cell membrane and can catalyze reactions [33]–[38], and many proteins form aggregates and amyloidal structures in conditions when they pass to intermediate states [39]–[42].

Herein we have tried to clarify which amino acid residues determine the stability of the molten globule state of apomyoglobin. On the one hand, it can be proposed that the formation of this intermediate state is affected by hydrophobic amino acid residues in the protein hydrophobic core since it is known that at the first stage of folding (of about several milliseconds) hydrophobic collapse of the polypeptide chain occurs [43]–[47]. On the other hand, we can suggest that the hydrophobic amino acid residues on the protein surface interacting with the solvent molecules also affect the molten globule state. The substitution of hydrophobic residues on the protein surface can have an effect on protein misfolding, i.e. decrease or increase the probability of formation of irregular hydrophobic contacts during protein folding. We have also postulated that the introduced disulfide bond can influence the molten globule state because the SS-bond should affect the mobility and compactness of the protein. By our hypothesis, the disulfide bond should be introduced on the protein surface so that the packing of the hydrophobic core of apomyoglobin would remain undamaged.

To verify the above proposals, we have studied four mutant forms of apomyoglobin with substitutions of hydrophobic amino acid residues on its surface and ten mutant forms of apomyoglobin with substitutions of amino acid residues in the hydrophobic core of this protein. In addition, we have analyzed the mutant form of apomyoglobin with the introduced disulfide bond on the protein surface.

## Results and Discussion

### Studies of Substitutions of Hydrophobic Amino Acid Residues

**Figure 1:**
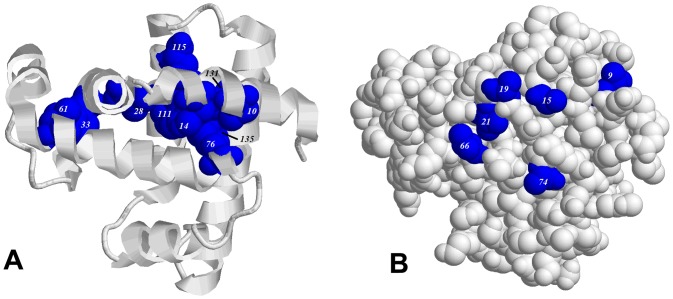
Choice of mutations in apomyoglobin. Three-dimensional structure of myoglobin (PDB-code: 1BZP) is shown with band model (A) and with balls models (B). Hydrophobic amino acid residues selected to substitute analine (A) and hydrophilic amino acid residues (B) are shown in blue color.

The following amino acid residues have been selected in the hydrophobic core of apomyoglobin: V10, W14, I28, F33, L61, L76, I111, L115, L131, and L135. They are large hydrophobic amino acid residues located in different structural elements of apomyoglobin (Fig. 1). Earlier we investigated the effect of these residues on the rates of refolding and unfolding of the apomyoglobin structure [11]. In this study, we analyze only the influence of single substitutions of hydrophobic amino acid residues on the stability of the molten globule state of apomyoglobin.

Hydrophobic amino acid residues on the protein surface were also chosen for substitutions. Based on the crystal structure of myoglobin (PDB-code: 1bzp) we selected residues that are maximally exposed to the aqueous environment and weakly interact with other amino acid residues of the protein. Six hydrophobic amino acid residues were selected to be substituted by hydrophilic ones (Fig. 1B). Four mutant proteins were examined: with substitutions of two (A15S and A19S), three (A15S, A19S, and V21T), four (L9D, A15S, A19S, and V21T) and six (L9D, A15S, A19S, V21T, V66T, and A74S) amino acid residues. Later the mutant proteins were designated as m2, m3, m4, and m6, respectively. Fig. 1 shows a three-dimensional model of apomyoglobin. As can be seen, the chosen amino acid residues on the surface of apomyoglobin are grouped on one side of the protein. We suggest that such positions of residues can enhance the effect of mutation on the molten globule state of apomyoglobin.

**Figure 2:**
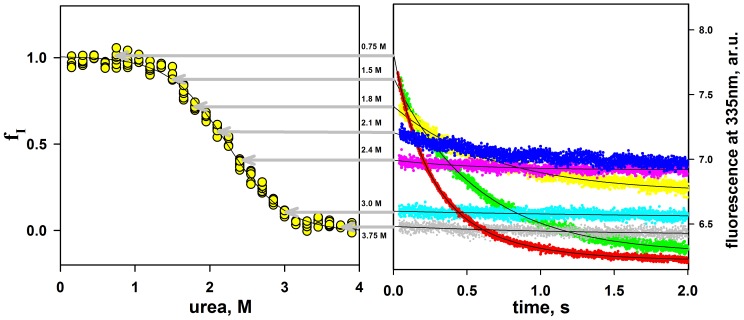
Refolding experiments. Right panel: Curves of refolding kinetics of apomyoglobin with substitutions A15S and A19S (m2) measured at different urea concentrations with the Trp fluorescence method (emission of 335 nm, excitation of 280 nm). The initial urea concentration was 6.0 M; numbers near the curves indicate final urea concentrations. Solid lines represent single-exponential approximations of the kinetics to zero time. Left panel: Population f_I_ of the molten globule state of protein m2 calculated from the burst-phase fluorescence amplitudes of refolding kinetics. The error of calculated f_I_ does not exceed the size of symbols.

We performed kinetic experiments on mutant forms of apomyoglobin refolding monitored by Trp fluorescence at 335 nm. For example, Fig. 2 (right panel) shows time-resolved courses of the Trp fluorescence changing during refolding of mutant form m2 of apomyoglobin (from 6 M urea to various final urea concentrations). It is seen that at zero time t_0_ the intensity values of Trp fluorescence at 335 nm are different. This is an indication that within the dead time of the instrument the intermediate state is accumulated. So, at final urea concentrations below 3 M, there are two consecutive refolding phases: The first phase (burst phase) occurs within the dead time of a stopped-flow instrument and is revealed by a jump-wise increase of fluorescence intensity, and the second phase is observed as a slow decrease of fluorescence intensity. At final urea concentrations above 3 M, there is only one fast phase, which manifests itself as a burst-like insignificant increase of fluorescence intensity. So, owing to the instrument dead time, it is only the result of the fast phase (i.e., the transition from the unfolded state to the kinetic intermediate state of apomyoglobin) that can be observed. After the protein, refolding is completed (i.e., time→∞) the fluorescence intensity values correspond to the equilibrium values. It should be noted that the kinetic intermediate *I* has a higher fluorescence intensity (at 335 nm) than that of the native *N* or unfolded state *U*. This property of the intermediate state is used to separate the kinetic transition *U*↔*I* from the transition *I*↔*N*. Since the slow phase of apomyoglobin refolding always leads to a decrease of fluorescence intensity, folding into the native state is believed to start from the intermediate state. At a given urea concentration M, the transient intermediate state population f_I_(M) can be calculated from the burst phase amplitude A(M) (see Materials and Methods, and [1]). Baryshnikova et al. [1] described in detail the approach allowing calculating the dependence of the population of the apomyoglobin intermediate state f_I_(M) on the urea concentration. The gist of the method is that the population of a rapidly formed intermediate state affects the amplitude of the subsequent slow kinetics of folding. For example, Fig. 2 (left panel) demonstrates the population of the molten globule state f_I_(M) of mutant form m2 of apomyoglobin calculated from the burst phase amplitude kinetic curves in Fig. 2 (right panel) according to Equation 5 (see Materials and Methods, and [1]).

**Figure 3:**
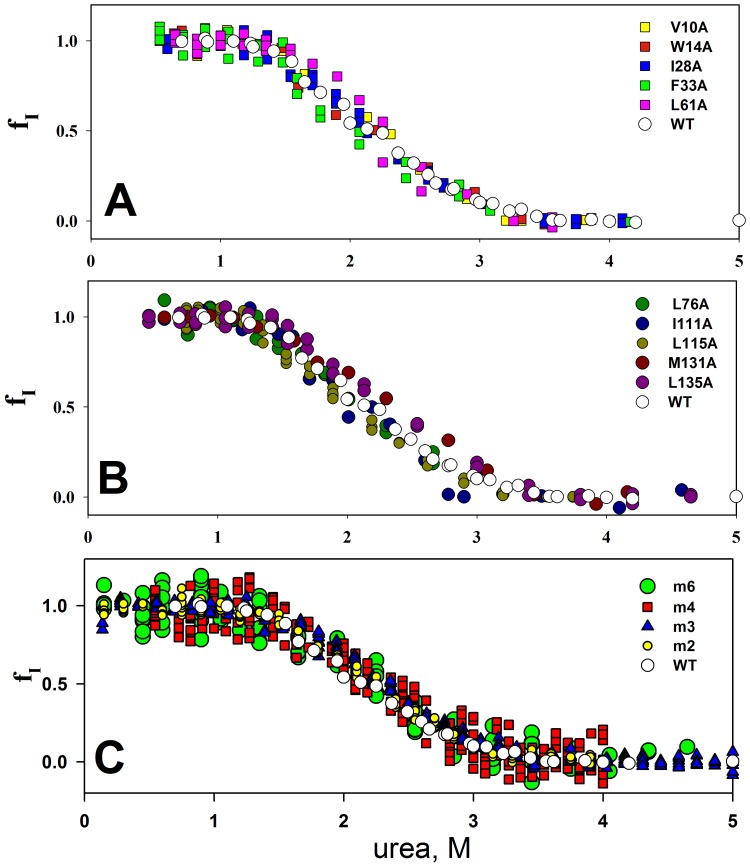
Population f_I_ of the molten globule state of apomyoglobin mutants. Dependency of the population f_I_ of the molten globule state versus urea concentration for apomyoglobin (WT) and its mutant forms with single substitutions of amino acid residues in the protein hydrophobic core (A and B) and with substitutions of amino acid residues on the protein surface (C): m2 – A15S, A19S; m3 - A15S, A19S, V21T; m4 – L9D, A15S, A19S, V21T; m6 – L9D, A15S, A19S, V21T, V66T, A74S.

**Figure 4:**
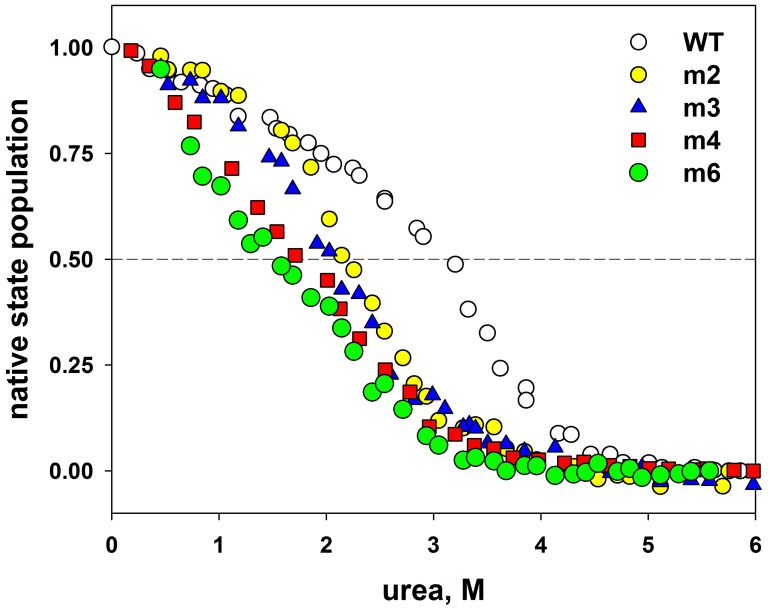
Urea induced equilibrium unfolding of apomyoglobin mutants. Equilibrium unfolding of apomyoglobin (WT) and its mutant forms m2 – A15S, A19S; m3 - A15S, A19S, V21T; m4 – L9D, A15S, A19S, V21T; m6 – L9D, A15S, A19S, V21T, V66T, A74S) measured with the method of Trp fluorescence. Native state population was calculated as a normalized value of the relation of fluorescence intensities at wavelengths of 320 nm and 380 nm (I_320_/I_380_).

The kinetics of refolding for all mutant forms of apomyoglobin were measured and the populations of the molten globule state were calculated. Fig. 3 shows dependencies of populations f_I_ of the molten globule state versus urea concentration for all mutant forms of apomyoglobin with substitutions of hydrophobic amino acid residues on the protein surface and in its hydrophobic core. Table 1 lists values of urea concentration corresponding to the midpoint of transition *MG*↔*U* (C_m,*MG↔U*_) for wild type apomyoglobin (WT) and its mutant forms. As can be seen, none of these substitutions have effect on the stability of the molten globule state. But all mutations change the stability of the native state of apomyoglobin. This can be concluded from the curves of equilibrium unfolding of the mutant forms of apomyoglobin. Fig. 4 demonstrates the curves of equilibrium unfolding for the four mutant proteins with substitutions of hydrophobic amino acid residues on the surface of apomyoglobin. The equilibrium unfolding study of the mutant forms of apomyoglobin with amino acid residue substitutions in the protein hydrophobic core was described in detail in our previous study [15], [48]–[49]. It should be noted that urea-induced equilibrium unfolding curves of apomyoglobin represent three-state transition [1]. This means that transition from native state *N* to unfolded state *U* contains two transitions *N*↔*MG* and *MG*↔*U*, which cannot be separated. Table 1 lists the data on equilibrium unfolding of the mutant forms of apomyoglobin with hydrophobic residue substitutions both on the protein surface and in its hydrophobic core. Table 1 shows that the urea concentration values corresponding to the midpoint of equilibrium unfolding C_m,*N↔U*_ differ for all mutant forms of apomyoglobin, and it is seen that all studied mutations destabilized transition *N*↔*U*. The urea concentration values corresponding to the midpoint of population of the molten globule state C_m,*MG↔U*_ are the same within the error. Thus, all the studied hydrophobic amino acid residue substitutions affected the native state of apomyoglobin to a different extent, but did not change the stability of the molten globule state. As C_m,*N↔U*_ is dependent on two transitions (*N*↔*MG* and *MG*↔*U*), the fact that for some mutants, e.g. L61A, m4 and m6, the C_m,*N↔U*_ values calculated from equilibrium unfolding (fig. 4), are lower than the C_m,*MG↔U*_ values calculated from population curves (Fig. 3) means that the transition from native state *N* to molten globule state *MG* proceeds at urea concentrations much lower than those upon transition from molten globule state to unfolded state *U*. This circumstance is not surprising and is observed for many proteins the unfolding of which results in accumulation of intermediate states. In other words, when the denaturant concentration increases, the protein first passes from native state *N* to intermediate state *I*, and then from intermediate state *I* to unfolded state *U*. It is evident that transition *N*↔*I* is less stable than transition *I*↔*U*. This takes place, for example, in some mutant forms of apomyoglobin studied in detail [11] as well as in carboxyanhydrase [14], lipase [50] and other proteins [51], [52].

**Table 1:** Parameters of equilibrium transitions *N*↔*U* and *MG*↔*U* for apomyoglobin and its mutant forms at pH 6.2, t = 11°C.

Apomyoglobin mutation	*N*↔*U*	*MG*↔*U*
	C_m,*N↔U*_ ±0.2 M	C_m,*MG↔U*_ ±0.2 M
WT	3.0	2.1
V10A	2.3	2.3
W14A	2.3	2.2
I28A	2.5	2.1
F33A	2.5	2.1
L61A	1.8	2.2
L76A	2.0	2.1
I111A	2.1	2.2
L115A	2.2	2.0
L131A	2.1	2.3
L135A	2.0	2.3
m2	2.2	2.2
m3	2.0	2.2
m4	1.7	2.2
m6	1.5	2.2

Thus, the above data show that substitutions of large hydrophobic amino acid residues for small alanine do not change the stability of the molten globule state of apomyoglobin. This is not striking because the molten globule state is a mobile state with nonspecific contacts [3], [53]–[55] and consequently substitutions of large hydrophobic amino acid residues for small alanine cannot change it indeed. However, the fact that substitution of a large number of hydrophobic residues (with the hydrophilic residues) on the protein surface does not affect the molten globule state was quite unexpected.

### Effect of the Disulfide Bond on the Energy Landscape of Apomyoglobin

The above investigations have allowed us to understand that hydrophobic amino acid residues substitutions (independently of where they are introduced in the protein) cannot influence the molten globule intermediate state. But which mutations can affect this state?

The molten globule state is a mobile state with nonspecific contacts [3], [53]–[55]. We have attempted to modulate these contacts using hydrophobic amino acid residue substitutions. Now let us try to affect the mobility of the molten globule state. We have proposed that the introduction of a disulfide bond can significantly change the polypeptide chain mobility and thereby will have an effect on the molten globule state.

**Figure 5:**
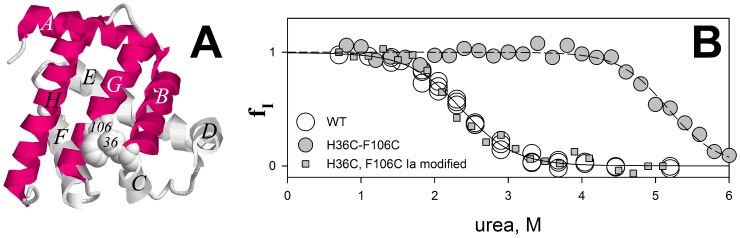
Effect of the SS-bond on apomyoglobin molten globule state stability. (A) Ribbon model of myoglobin. Letters A through H denote α-helices. Amino acid residues His36 and Phe106 replaced by cysteine residues are shown three-dimensionally. α-Helices that are the first to fold (see the text) are shown in red color. (B) Dependency of the population f_I_ of the molten globule state versus urea concentration for apomyoglobin (○), its mutant form with SS-bond between amino acids 36 and 106 (•) and the protein mutant form with cysteine residues modified by iodoacetamide (▪).

To simulate an SS-bond in apomyoglobin it is necessary to take into account several facts. Myoglobin has been crystallized only in a holo-form (with the heme). In apomyoglobin, α-helix F (see Fig. 5) is unfolded [56]. It is known from the NMR studies that α-helices A, B, G, and H are formed at the earliest stages of apomyoglobin folding [56]. Based on all this, in order to introduce SS-bond we have searched for amino acid residues neighboring on the surface of myoglobin located on α-helices A, B, G, and H. We have chosen His36 and Phe106. The distance and mutual orientation of these amino acid residues are appropriate for the formation of an SS-bond when they are substituted by cysteine residues.

Investigations of the mutant form of apomyoglobin with the double substitution (H36C and F106C) were performed under the following conditions: when the SS-bond was oxidized and an SS-bridge was formed, and when the SS-bond was disrupted upon modification of free SH-groups with iodoacetamide. Inasmuch as the SS-bond was designed on the surface of apomyoglobin, under condition when the SS-bond is disrupted the protein structure would not strongly differ from that of the wild-type protein. For the mutant form of apomyoglobin with the double substitution (H36C and F106C), we measured the refolding kinetics and calculated the population of the molten globule state. Fig. 5 shows the populations of the molten globule state for wild-type apomyoglobin and its mutant H36C F106C with an SS-bond and without it.

Fig. 5B shows that the SS-bond has strongly affected the stability of the molten globule state of apomyoglobin since the population plot is shifted towards higher urea concentration values by almost two and a half moles. In this case, the same plot for the protein with H36C and F106C substitutions but with cysteine residues modified by iodoacetamide is completely compatible with the plot for the wild-type protein.

Thus, it can be concluded that it is *the introduction of an SS-bond between amino acid residues 36 and 106 that affects the intermediate state of apomyoglobin rather than substitutions of amino acid residues on its surface*. It is fascinating to understand how the SS-bond has influenced the entire protein energy landscape.

For the mutant form of apomyoglobin with the double substitution (H36C and F106C) and the oxidized SS-bond, the kinetics of refolding and unfolding in the presence of various denaturant concentrations was measured using the method of Trp fluorescence. Based on approximation of the kinetic curves, we estimated rate constants of refolding/unfolding and obtained a chevron plot. A similar plot was obtained for the mutant protein with the introduced cysteine residues modified by iodoacetamide. This plot is identical with the plot for the wild-type protein (data not given). This confirms that it is the disulfide bond, which affects the energy landscape (it will be discussed below) rather than amino acid residue substitutions H36C and F106C.

**Figure 6:**
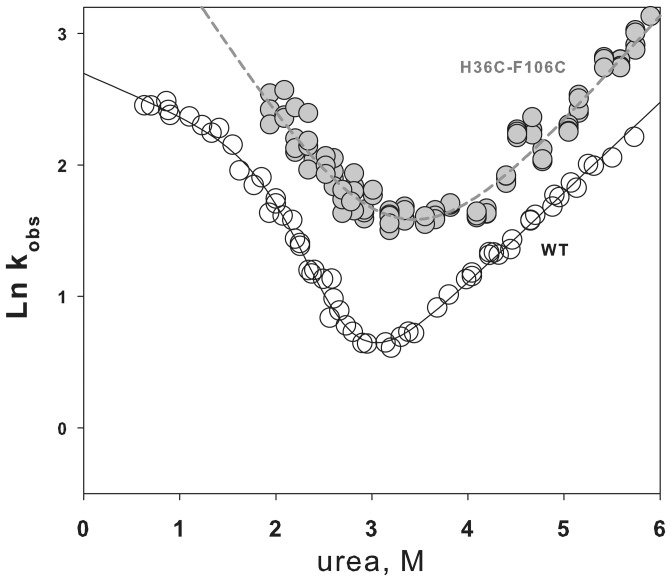
Chevron plots. Chevron plot for wild type apomyoglobin WT (○) and its mutant form with the introduced SS-bond between amino acid residues 36 and 106 (H36C-F106C) (•).

Fig. 6 demonstrates a chevron plot for the wild-type apomyoglobin and its mutant form with the SS-bond between amino acid residues 36 and 106. It is seen that the SS-bond has affected both the folding branch and the unfolding branch of the plot. The folding branch of the chevron plot for apomyoglobin with the SS-bond has changed mainly due to stabilization of the molten globule state (Fig. 5). By using formulas, 1–3 (see Materials and Methods), one can estimate free energies of all states of apomyoglobin. However it is impossible to calculate the height of the energy barrier between the molten globule and unfolded states because protein transition from one state to the other takes less than 5 milliseconds and cannot be measured using the stopped-flow device (see Materials and Methods).

**Figure 7:**
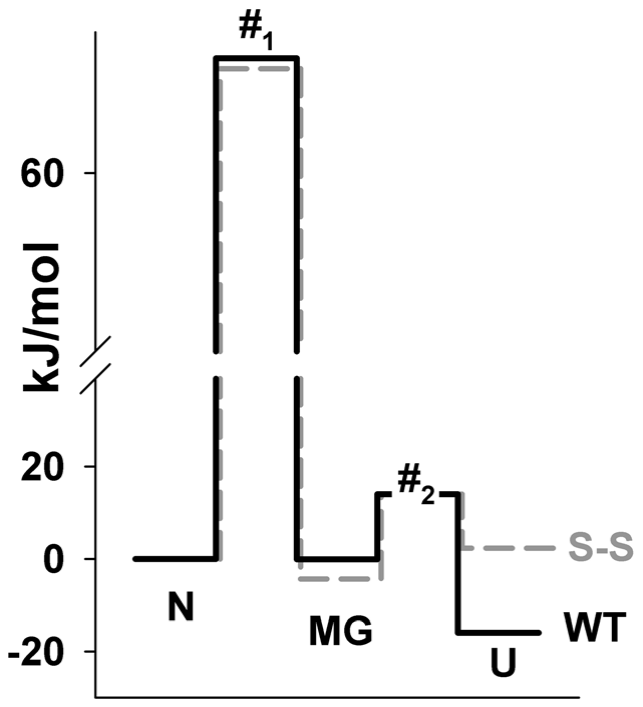
Effect of the SS-bond on apomyoglobin free energy profiles. Free energy profiles estimated for apomyoglobin (WT) and its mutant with a disulphide bond (S-S) between amino acid residues 36 and 106 in 4.3 M urea at pH 6.2, 11°C. N, protein native state; U, protein unfolded state; MG, Intermediate state of a molten globule type; #_1_, protein N↔MG transition state; #_2_, protein U↔MG transition state, the height of this energy barrier can not be determined experimentally because the U↔MG transition proceeds during the dead time of the instrument. The error of calculated free energy levels does not exceed 0.5 kJ/mol.

Fig. 7 shows profiles of free energies for apomyoglobin and its mutant form with the SS-bond estimated from the chevron plot in Fig. 6. One should remember that it is impossible to estimate absolute values of free energies of different states of the protein. We can estimate only the change in the free energy upon transition from one state to the other [21], [31], [57]. In other words, it is possible to estimate how energy levels are located in a protein relative to each other, but it is not always clear how these energy levels of different proteins can be compared. That is why when energy profiles of different proteins or their mutant forms are compared, there is free will in choosing the “reference point” (zero on the energy scale). The choice of such a “reference point” is based usually on additional suppositions. As a rule, it is accepted that free energies of unfolded states of proteins are equal and that unfolded conformations of different proteins are the same and represent statistical coils [1], [24], [31], [58]. The SS-bond introduced into the protein changes the conformation of the protein unfolded state [59]–[61]. Therefore, the disulfide bridge inserted in apomyoglobin has undoubtedly changed the entropy component of the free energy of the unfolded state of this protein [62]; therefore, it would be more correct to level the energy free profiles of apomyoglobin and its mutant form with the SS-bond by the energy of native states of these proteins. This means that to make the comparison more convenient we have attributed the zero value of the free energy in Fig. 7 to the protein native states. Another important moment that ensues from the impossibility to estimate absolute free energies values for different states of the protein is as follows. In the analysis of intricate energy schemes, it is impossible to estimate the stability of a definite state of protein; it is probable to estimate its stability relative to the other state of protein. So it is more correct to speak about the stability of transition between the two states rather than the stability of the state.

To understand the details of the influence of the SS-bond on apomyoglobin, using Fig. 7 it can be analyzed how the mutation affected the mutual arrangement of different protein states. For example, it can be seen that the SS-bond has destabilized transitions between the native state *N* and the molten globule state *MG*. In this case the SS-bond stabilized the transition between the molten globule state *MG* and the unfolded state *U*. The SS-bond had a weak effect on the transition state *#_1_*. This can be concluded from the weak influence of the SS-bond on the unfolding rates of the native state of apomyoglobin (see the unfolding branches of the chevron plot in Fig. 6) rather than from the change in the free energy profile.

## Conclusions and Speculations

The above allows us to make the following conclusions.

1. Single substitutions of hydrophobic amino acid residues on the surface and in the hydrophobic core of apomyoglobin do not affect the transition between its unfolded state *U* and molten globule state *MG*. This is evidence that the stability of the molten globule state of apomyoglobin does not change.
2. Introduction of the disulfide bond on the surface of apomyoglobin destabilized the *N* ↔ *MG* transition and stabilized the *MG* ↔ *U* transition. This means that the molten globule state of apomyoglobin was stabilized (its free energy decreased) as compared to the native and unfolded states. Though only one SS-bond was analyzed, the result obtained demonstrates that there is a principal possibility to affect the molten globule state via mutations that change the polypeptide chain compactness.

The analysis of the energy landscape of apomyoglobin with the introduced SS-bond permits us to make one more conclusion.

3. A decrease in the free energy of the intermediate state (with introduction of an SS-bond) in apomyoglobin leads to relative destabilization of the native state which shows that the transition between the native and intermediate states begins at lower concentrations of the denaturant.

**Figure 8:**
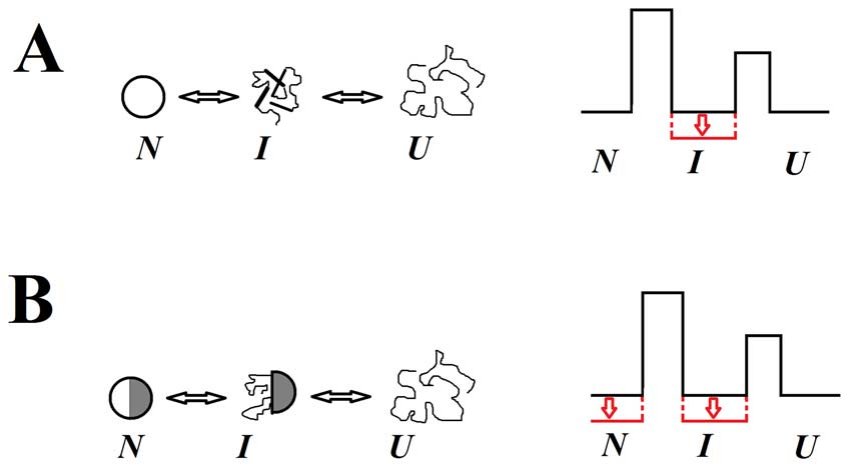
Energy schemes for proteins with different types of intermediate state and schematic representation of different states of such proteins. **A**, Scheme for a protein in which native and intermediate states differ in their properties. In this case it is possible to choose the mutation that will act selectively on one of the protein states, for example, on the intermediate state (shown in red color). **B**, Scheme for a protein in which the intermediate state is a partially “destroyed” native state. In this case states *I* and *N* are stabilized by similar interactions and the mutation affecting state *I* will also affect state *N*. This effect (decreasing free energy for *I* and *N*) is shown in red. *N*. *I* and *U* are native, intermediate and unfolded states of the protein, respectively.

At first sight, this conclusion may seem trivial. Indeed, if the free energy of the intermediate state in the energy scheme of any three-state protein (for example, see the scheme in Fig. 8A) is decreased by introduction of the mutation, the transition between the native *N* and intermediate states *I* would be destabilized while the transition between the intermediate *I* and unfolded states *U* would be stabilized. But such behavior of energy levels in a definite protein means that the native state *N* and the intermediate states *I* are stabilized by different interactions independent of each other.

Let us take an opposite situation when the intermediate state *I* is a partially unfolded native state *N* (Fig. 8B). In this case the native and intermediate states are similar, are stabilized by similar interactions, and the native state is an “overbuild” intermediate state. Consequently, the stabilization of the intermediate state (the free energy decrease) should also result in stabilization of the native state (the free energy decrease of N state), whereas the destabilization of the intermediate state should lead to destabilization of the native state. This is shown schematically in Fig. 8B.

The experimental data obtained for apomyoglobin are compatible with the scheme in Fig. 8A, thus supporting the proposal that completely different interactions are crucial for stabilization of the native and molten globule states of apomyoglobin. This is corroborated both by the analysis of substitutions of hydrophobic amino acid residues and by the introduction of an SS-bond on the protein surface.

Could the results of our studies be generalized? Should the above conclusions be taken in studying other proteins? Every investigator is justified to decide oneself. But if in our future studies we have to influence the stability of the molten globule state of a small globular protein using the minimal number of mutations, in any case we *will not* use substitutions of hydrophobic amino acid residues but will prefer to introduce an SS-bond.

### Why Can the SS-bond Destabilize Protein?

Many researchers try to introduce an SS-bond to stabilize proteins. And some SS-bond do stabilize the protein, whereas others destabilize it [63]–[66]. All cases of destabilization are most often explained by an inadequate design of the SS-bond suggesting that the SS-bond causes tension in the protein structure and thus destabilizes it. The above described effect of the SS-bond on apomyoglobin shows that when the SS-bond decreases the free energy of the intermediate state, this should lead to destabilization of the transition between the native and intermediate states. In this case, protein denaturation will begin at lower denaturant concentrations, melting can occur at lower temperatures, and the protein activity can change, i.e. we will notice many features typically observed upon destabilization of two-state proteins.

That is why is in a two-state protein the SS-bond is designed correctly without steric tension, it will always lead to protein stabilization (because it has an effect on the entropy component of the free energy of the unfolded state [62]). In a multistate protein, the introduced SS-bond can lead to absolutely different and frequently unpredictable results. And this is not connected with an “incorrect” design of the SS-bond, but with our misunderstanding of the nature of the formation of protein intermediate states.

## Materials and Methods

### Protein chemistry

Plasmids with mutant apoMb genes were constructed by polymerase chain reaction with the plasmid pET17 (a kind gift of P.E. Wright) containing the sperm whale apoMb gene, appropriate primers, and using a QuikChange (Stratagene, La Jolla, CA) kit [67]. WT apoMb and its mutants were isolated and purified as described previously [1], [67] after expression of appropriate plasmids in *Escherichia coli* BL21 (DE3) cells. The protein purity was checked by SDS/PAGE electrophoresis. The protein concentration was determined spectrophotometrically from absorbance at 280 nm using the extinction coefficient E^1%^_1 cm_  = 0.80 for all samples [68], [69], except for one Trp-lacking mutant with substitution W14A; for this one, the used extinction coefficient was determined from nitrogen-based measurements according to Jaenicke [69] and appeared to be E^1%^_1 cm_  = 0.56 [4].

Disulfide bond formation was performed as follows. The pure protein was dissolved in 0.2 M Tris-HCl, pH 7.5, 0.2 M NaCl, 1 mM EDTA to the protein concentration of 5 mg/ml. The protein was oxidized by addition of oxidized and reduced glutathione to final concentrations of 10 mM and 2 mM, respectively. After 24 h incubation at room temperature, glutathione was removed with a PD-10 Desalting column. Then the quantity of free SH groups was defined using the Ellmans reagent [70].

### Selection and Substitution of Amino Acids in Apomyoglobin

The principle used for selection of hydrophobic amino acid residues in the apomyoglobin core was described in our previous publications [11]; in general, they are conservative amino acid residues that form a hydrophobic core of the protein. Among the hydrophobic amino acid residues on the surface of apomyoglobin we selected those which are maximally exposed to the aqueous environment. During this, we substituted the amino acid residues for hydrophilic ones following the principle of minimal differences in amino acids. For example, alanine was substituted for serine because the both amino acids differ only in one OH group.

### Equilibrium Studies of Urea-induced Denaturing Transitions

All fluorescence measurements were carried out at 11°C; 0.01 M sodium acetate, pH 6.2, was used as a buffer system. Such experimental conditions (temperature and pH) were chosen because previously these conditions were used to study in detail wild-type apomyoglobin and to characterize its intermediate state [1]. It should be added that pH 6.2 is optimal for stability of the native state of apomyoglobin and the temperature of 11°C is optimal for performing kinetic experiments. The details of choosing the experimental conditions are described elsewhere [1], [11], [71]. Equilibrium protein Trp fluorescence measurements were performed using a Cary Varian 100 spectrofluorimeter (Australia) with standard 1 cm path-length quartz cuvettes. The excitation wavelength was 280 nm. The emission spectra were recorded between 300 and 500 nm at the protein concentration of 0.1 mg/ml.

### Kinetic Studies

Kinetic measurements were taken using a spectrofluorimeter Chirascan Spectrometer (Applied Photophysic, UK) equipped with a stopped-flow attachment. The excitation wavelength was 280 nm, and emission spectra were recorded using a 320-nm cut-off glass optical filter. The dead time of the equipment was <20 ms. All measurements were taken at 11°C, pH 6.2. Because apomyoglobin folds quite rapidly at room temperature, a lower temperature should be used to slow down the reaction rate. On the other hand, the possibility of cold denaturation of this protein previously reported by Griko et al. [72], [73] made us choose 11°C for our experiments [1]. The final protein concentration was 0.03 mg/mL. The initial urea concentration was 6.0 M for refolding experiments, and 0.0 M for unfolding ones. The initial protein solution was mixed (1∶1) with a buffer of various urea concentrations using the stopped-flow attachment. All obtained kinetic curves were well described (after the dead-time burst phase) by a single-exponential approximation. Kinetic data at the same conditions were used repeatedly ten times for each point, then the experimental data set was averaged and the averaged data were used to estimate the parameters (amplitude *I*_335nm_ and *k*_obs_).

### Reversibility of Folding of Apomyoglobin and Its Mutant Forms in Equilibrium and Kinetic Experiments

Reversibility of folding of apomyoglobin and its mutant forms was checked using different methods. Equilibrium studies were performed both of the native and unfolded states of the protein: apomyoglobin in the native state was added to the samples with different urea concentrations. So the protein unfolding took place in each of the samples. Apomyoglobin in the unfolded state (6 M urea) was added to the samples with different urea concentrations in a similar way, and the protein folding took place in each of the samples. After 10 min incubation of the samples at 11°C their Trp fluorescence spectra were measured. The fact that the fluorescence spectra of the samples depend only on the urea concentration and do not depend on whether the folding and unfolding processes took place in them, support the reversibility of the folding/unfolding processes in apomyoglobin. In the kinetic experiments, the rate constants of folding and unfolding are compatible in the middle of chevron plots. This is an additional confirmation that the folding “pathway” and unfolding “pathway” of apomyoglobin coincide. Detailed explanations and corroborative plots were published in our earlier papers [1], [48].

### Analysis of Equilibrium Transitions and Population Curves of the Molten Globule State

Curves of equilibrium unfolding of apomyoglobin and its mutant forms measured by the method of Trp fluorescence (Fig. 4) yield information on both the transition of proteins from the native state to the molten globule state (*N*↔*MG*) and transition from the molten globule state to the unfolded state (*MG*↔*U*). Unfortunately it is impossible to separate the two transitions and to estimate their individual parameters. That is why the only parameter we can estimate from curves of equilibrium unfolding is the urea concentration corresponding to the middle of the appropriate curve C_m,*N↔U*_. C_m,*N↔U*_ is the concentration of urea when the normalized experimental curve makes 0.5. With account of the error in determining the urea concentration and the scattering of experimental curves, the C_m,*N↔U*_ values can be determined with an accuracy of 0.2 M. These values are given in Table 1.

The curves of population of the molten globule state (Fig. 3) were used to estimate C_m,*MG↔U*_ values (see Table 1). Inasmuch as these curves reflect the transition between the two states *MG*↔*U*, the following equation can be used to describe them:
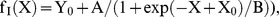
where X is the concentration of urea and Y_0_, A, B, and X_0_ are fitting parameters. Here parameter X_0_ determines the position of the middle of the sigmoid curve, i.e. C_m,*MG↔U*_  = X_0_. Fitting of the experimental data with the above equation allows us to more correctly determine the C_m,*MG↔U*_ value. However, with the scattering of the experimental points, the accuracy of determining this parameter does not exceed 0.2 M either.

### Analysis of the Chevron Plot

Rate constants of the transition over the rate-limiting free energy barrier between *I* and *N* (*k_NI_* for the *N→I* transition and *k_IN_* for *I→N*) are considerably less than the *I↔U* rates *k_UI_* and *k_IU_* (here, about 1 s^−1^ and 10^−3^ s^−1^, respectively), thereby the experimentally observed rate constants can be described by the equation [20], [74]

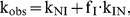
(1)where f_I_ is an intermediate state population proportional to the amplitude of the burst-phase of refolding kinetics (Fig. 2).

### Plotting of Free-energy Profiles

The rate constants of the fast *I↔U* transition (*k_UI_* and *k_IU_*) cannot be measured experimentally because this event occurs within the stopped-flow dead time. Nevertheless, relative positions of free energies F for *U, I, N* states (*F_U_, F_I_, F_N_*), and for transition state *#_1_* (*F_#1_*) can be estimated over the entire range of urea concentrations using the experimentally measured rate constants for protein folding/unfolding (*k_obs_*) and percentage of population of the *I* state (*f_I_*).

*F_I_-F_U_* can be obtained, at various urea concentrations *M*, as
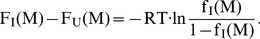
(2)
*F_N_-F_I_* can be obtained from the *N↔I* two-state transition [24] as
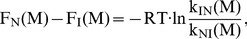
(3)where *k_NI_(M)* is the unfolding rate and *k_IN_(M)* is the refolding rate constants extrapolated to urea concentration *M*.

The rate constant *k_NI_* is determined by the *F_TS_-F_N_* difference (see the transition state theory [24], [75]) so *F_TS_-F_N_* can be obtained as:
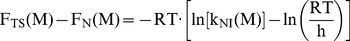
(4)where
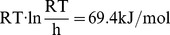
 is the constant at 11°C (284K).

### Determination of the Population of the Kinetic Intermediate

The kinetic intermediate (molten globule state) population among the nonnative states, i.e., *f_I_(M)  =  [I]/([I] + [U])*, where *[I]* and *[U]* are concentrations of the *I* and *U* states, was determined at various urea concentrations *M* from the burst phase fluorescence amplitude, 
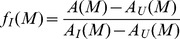
(5)where *A_U_(M)* and *A_I_(M)* are the integral fluorescence intensities for *U* and *I* states, and *A(M)* is the integral fluorescence intensity achieved as a result of the first, burst *(U → I)* phase in the *(U → I)* transition in protein folding at the given final urea concentration *M*.
